# Neurophysiology of Sleep and Wakefulness: Basic Science and Clinical Implications

**DOI:** 10.2174/157015908787386050

**Published:** 2008-12

**Authors:** Jonathan R.L Schwartz, Thomas Roth

**Affiliations:** 1 Integris Sleep Disorders Center of Oklahoma, University of Oklahoma Health Sciences Center, Oklahoma City, OK, USA; 2 Henry Ford Hospital Sleep Disorders and Research Center, Detroit, MI, USA

**Keywords:** Excessive sleepiness, neurobiology, arousal system, sleep-wake states, circadian rhythm, sleep disorders.

## Abstract

Increased attention to the prevalence of excessive sleepiness has led to a clear need to treat this symptom, thus reinforcing the need for a greater understanding of the neurobiology of sleep and wakefulness. Although the physiological mechanisms of sleep and wakefulness are highly interrelated, recent research reveals that there are distinct differences in the active brain processing and the specific neurochemical systems involved in the two states. In this review, we will examine the specific neuronal pathways, transmitters, and receptors composing the ascending arousal system that flow from the brainstem through the thalamus, hypothalamus, and basal forebrain to the cerebral cortex. We will also discuss the mutually inhibitory interaction between the core neuronal components of this arousal system and the sleep-active neurons in the ventrolateral preoptic nucleus, which serves as a brainstem-switch, regulating the stability of the sleep-wake states. In addition, we will review the role of homeostatic and circadian processes in the sleep-wake cycle, including the influence of the suprachiasmatic nucleus on coordination of sleep-wake systems. Finally, we will summarize how the above processes are reflected in disorders of sleep and wakefulness, including insomnia, narcolepsy, disorders associated with fragmented sleep, circadian rhythm sleep disorders, and primary neurological disorders such as Parkinson’s and Alzheimer’s diseases.

## INTRODUCTION

Sleep loss and disorders of sleep/wake function are among the most common health problems reported in the United States. The estimated prevalence of syndromes of sleep-wake disorders in the US is about 50 to 70 million [75], and those who suffer from chronic sleep disorders have impaired daily functioning, compromised health status, and diminished quality of life [41]. As a consequence, during the past decade, much attention has been focused on the need to treat these conditions and thus on the neurophysiology of sleep and wakefulness. This research has led to a heightened understanding of the mechanisms regulating these behavioral states and the pathways, transmitters, and receptors involved in the sleep-wake cycle [14,17,34,50,53,59,68,98,99,107]. It is now apparent that the neural circuitry underlying the regulation of sleep and wakefulness is discrete for each state yet interdependent; the very arousal systems that are inhibited by sleep-promoting neurons also serve to disrupt these same sleep processes to return the body to a wakeful state [99]. 

Alertness and associated forebrain and cortical arousal are mediated by several ascending pathways with distinct neuronal components that project from the upper brain stem near the junction of the pons and the midbrain [33]. One pathway innervates the thalamus, and the second extends into the posterior hypothalamus and forebrain. Key cell populations of the ascending arousal pathway include cholinergic, noradrenergic, serotoninergic, dopaminergic, and histaminergic neurons located in the pedunculopontine and laterodorsal tegmental nucleus (PPT/LDT), locus coeruleus, dorsal and median raphe nucleus, and tuberomammillary nucleus (TMN), respectively. Projections from these various cell groups fire in a characteristic pattern to promote arousal. However, every 24 hours the arousal system is inhibited during sleep by sleep-active γ-aminobutyric acid (GABA)-ergic and galaninergic neurons of the ventrolateral preoptic nucleus (VLPO). The interaction between the VLPO and the branches of the ascending arousal pathway is mutually inhibiting, functioning much like an electrical “on-off” switch, enabling the body to maintain a stable state of wakefulness and sleep [34,59,98]. Normally, this “sleep-wake switch” design ensures stability between sleep and wakefulness while promoting rapid transitioning between the two behavioral states. Sleep disorders represent a pathology of this switch, which causes individuals to suffer from state instability, with wake intruding into sleep and/or sleep intruding into wake. 

In this review, we will describe the arousal and sleep-promoting neural pathways, looking closely at the brain circuitry, neurotransmitters, and chemical substrates involved in sleep control. In addition, we will examine the two processes engaged in the homeostatic and circadian regulation of sleep [1,12]. Finally, the review will describe how the aforementioned physiological and neurochemical processes are manifested in conditions such as insomnia, narcolepsy, disorders caused by fragmented sleep, circadian rhythm disorders, and impaired sleep due to primary neurological disease, offering potential new targets for pharmaceutical treatment. If this research is successful, it could, over time, improve the considerable burden of disease associated with these debilitating sleep disorders. 

## THE ASCENDING AROUSAL SYSTEM INDUCES WAKEFULNESS

Contemporary models of the wake-sleep regulatory system are based on the seminal research conducted by von Economo, Moruzzi, and Magoun. (A detailed review appears in Saper 2005 [99].) In 1930, von Economo reported that a viral illness known as encephalitis lethargica was caused by lesions of the posterior hypothalamus and rostral midbrain [122]. Consequently, he hypothesized that wakefulness is mediated by an ascending arousal system beginning in the brainstem, which remains active following midbrain interruption of the classical sensory pathways. 

**Fig. (1):**
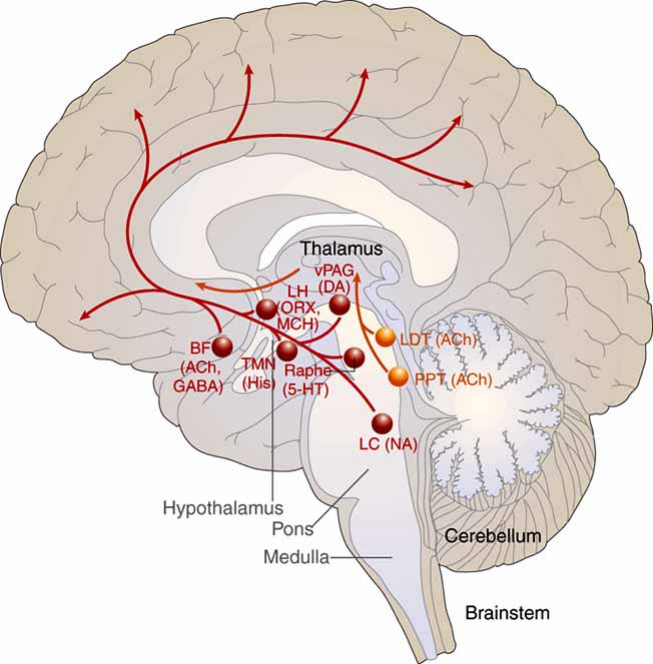
A schematic drawing showing key components of the ascending arousal system. Adapted from Saper 2005, pg 1258 [99].

Nearly two decades later, Moruzzi, Magoun, and colleagues confirmed that waking behavior is indeed maintained by an “ascending reticular activating system,” originating in the upper brainstem adjacent to the junction of the pons and midbrain and continuing on to the diencephalon, where it separates into two branches [72,109]. In fact, it is now known that the ascending arousal system contains two major branches, each comprising discrete cell populations and neurotransmitters [98] (Fig. **1**). The first branch innervates the thalamus, activating relay neurons and reticular nuclei essential for thalamocortical transmission. Two cholinergic structures in the brainstem and basal forebrain serve as the origin of these projections to the principal thalamic nuclei – the PPT/LDT nuclei [40]. PPT/LDT neurons are most active during wakefulness and rapid eye movement (REM) sleep and discharge more slowly during non-REM (NREM) sleep, a period when cortical activity is reduced [99]. Transmission to the reticular nucleus of the thalamus is of particular importance, as the site functions as a gating mechanism that can block the generation of thalamocortical rhythms and promote a state of excitability and wakefulness [57]. Other projections from the upper brainstem to the midline and intralaminar thalamic nuclei, which include the reticular formation, the parabrachial nucleus, and the monoaminergic systems (discussed below), are also believed to be involved in cortical arousal [48]. 

The second branch of the ascending arousal system projects into the lateral hypothalamus, basal forebrain, and the cerebral cortex [45,96,98]. It comprises a number of monoaminergic cell populations, including noradrenergic neurons of the locus coeruleus, serotoninergic dorsal and median raphe nuclei, dopaminergic neurons of the ventral periaqueductal grey matter, and the histaminergic TMN. Several additional cerebrocortical afferents have been identified: lateral hypothalamic peptidergic neurons, which contain melanin-concentrating hormone or orexin/hypocretin, and basal forebrain nuclei, which contain acetylcholine or GABA [99]. Neurons in these monoaminergic systems have broad action potentials, discharging most rapidly during wakefulness, slowing during NREM sleep, and showing little activity during REM sleep [8,33,110]. A similar pattern was reported in orexin/hypocretin neurons of the lateral hypothalamus [32, 63]. In contrast, melatonin-concentrating neurons, which play an important role in REM homeostasis, are strongly active during REM sleep [119], and cholinergic neurons of the basal forebrain discharge at maximal rates during both REM sleep and active waking [49]. Lesions along this second branch are associated with narcolepsy and other sleep disturbances in rats [37], with the loss of hypocretin cells, in particular, contributing to the difficulty in maintaining arousal and the loss of muscle tone during cataplectic attacks [63]. 

In sum, cholinergic neurons, monoaminergic cell populations, and orexin/hypocretin nuclei of the lateral hypothalamus located along the two branches of the ascending arousal system, discharge in a stereotypical and coordinated manner to promote cortical arousal, with each making unique, though overlapping and redundant, contributions to achieve and sustain wakefulness. During sleep, these circuits are blocked by neurons of the VLPO. 

## THE VLPO AND THE SLEEP STATE

**Fig. (2):**
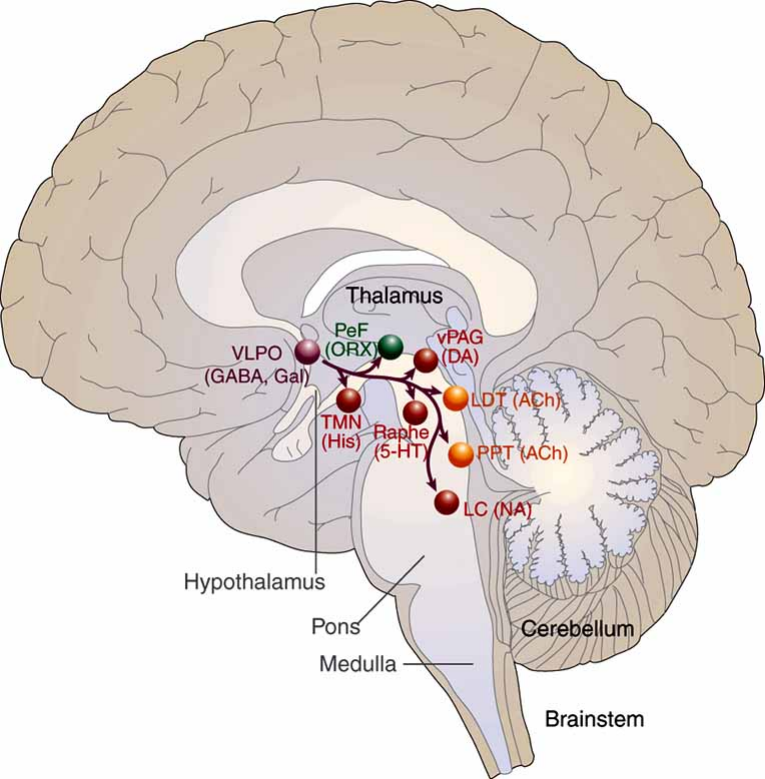
A schematic drawing showing primary projections of the VLPO to the main components of the ascending arousal system. Adapted from Saper 2005, pg 1258 [99].

Following experiments by McGinty and colleagues, which demonstrated that lesions in the basal forebrain suppressed sleep in cats [58], Sherin *et al.* determined that a group of ventrolateral preoptic neurons is specifically activated during sleep [107]. Neurons of the VLPO form a dense cluster and also extend more diffusely to innervate the monoaminergic systems in the hypothalamus and brainstem that participate in the modulation of cortical arousal (Fig. **2**). VLPO efferents contain the inhibitory neurotransmitters GABA and galanin, and have been shown to play a central role in the mammalian brain in quieting the ascending monoaminergic arousal system during sleep [36,106].

Experiments in different animal species have indicated that injury to the VLPO cluster and extended VLPO decreases NREM and REM sleep, respectively [51,53]. Neurons of the extended VLPO connect with pontine sites implicated in REM sleep gating – the LDT, dorsal raphe nucleus, and locus coeruleus [51], whereas the VLPO cluster provides output to histaminergic neurons of the TMN, which, as noted previously, are active during waking, reduce firing during NREM sleep, and cease discharge during REM sleep [42, 50].

Afferents from the components of the monoaminergic arousal system also connect with the VLPO [18]. Noradrenaline and serotonin released by axons from the locus coeruleus and median raphe nuclei, respectively, inhibit VLPO neurons in recordings of cells in hypothalamic slices [34], as do GABA [16] and galanin [46] produced by TMN neurons. (VLPO neurons do not appear to have receptors for histamine.) Thus, the reciprocal inhibitory interaction of sleep-promoting VLPO neurons and the noradrenergic, serotoninergic, and cholinergic waking systems to which they project establishes a remarkable dynamic, in which the VLPO is down-regulated by the very arousal systems it blocks during sleep [34,10].

## THE BRAINSTEM CONTROL OF STATE STABILITY

**Fig. (3):**
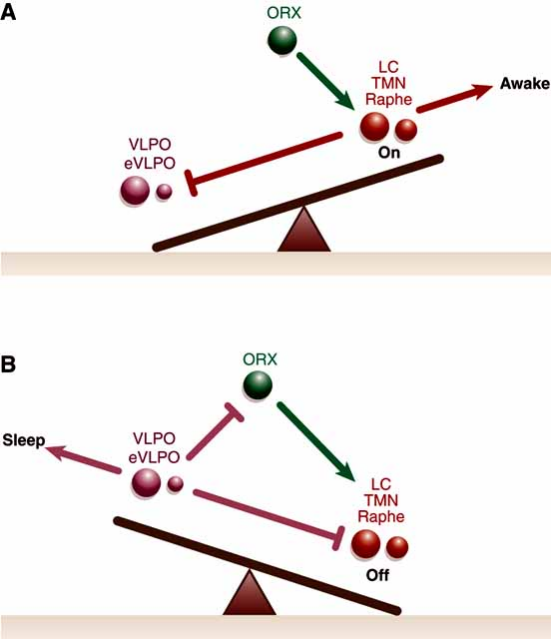
A schematic diagram of the flip-flop switch model. Adapted from Saper 2005, pg 1259 [99].

The reciprocal inhibitory exchange between the major ascending monoaminergic arousal groups and the sleep-inducing VLPO acts as a feedback loop; when monoamine nuclei discharge intensively during wakefulness, they inhibit the VLPO, and when VLPO fire rapidly during sleep, block the discharge of the monoamine cell groups [98]. This relationship is described as a bistable, “flip-flop” circuit, in which the two halves of the circuit strongly inhibit each other to produce two stable discharge patterns – on or off (Fig. **3**). Intermediate states that might be partially “on and off” are resisted. This model helps clarify why sleep-wake transitions are relatively abrupt and mammals spend only about 1% to 2% of the day in a transitional state [99]. Hence, changes between sleep and arousal occur infrequently and rapidly. As will be described below, the neural circuitry forming the sleep switch contrasts with homeostatic and circadian inputs, which are continuously and slowly modified [98].

Despite the bistability of the on/off feedback loop, if either side is weakened or injured, unwanted instability can occur during both sleep and wake states, irrespective of which side is damaged. For instance, animals with VLPO lesions experience a 50% to 60% reduction in NREM and REM sleep time and wake up frequently during their sleep cycle [52]. Rapid sleep-wake cycling also is common in the elderly [6], who have fewer VLPO neurons [36]. These findings suggest that when the self-reinforcing properties of the circuitry are weakened, individuals shift back and forth between sleep and wakefulness more frequently as well.

## PROMOTING SLEEP-STATE STABILITY

Orexin (hypocretin) is a neuropeptide produced by the neuronal cluster in the posterior portion of the lateral hypothalamus [24,94]. Neurons containing orexin innervate the major nuclei implicated in sleep regulation. Specifically, orexin-1 receptors are found in the locus coeruleus, orexin-2 receptors in the TMN, and both types in the median raphe nuclei and mesopontine reticular formation [54]. In contrast, orexin/hypocretin neurons only modestly affect the VLPO [18,95,128]. Therefore, it is possible that orexin/hypocretin may promote wakefulness by up-regulating monoaminergic neuronal populations. 

This hypothesis has been confirmed in animals and humans. Experiments using orexin-receptor knockout mice produce behavioral symptoms and electroencephalographic results strikingly similar to those of humans with narcolepsy, which is characterized by sleep/wake as well as REM sleep dysregulation [17]. Similarly, humans who have narcolepsy with cataplexy have been shown to have few orexin neurons in the lateral hypothalamus and low orexin levels in the cerebrospinal fluid [61,86,113]. Fos expression in orexin/hypocretin neurons was correlated positively with wakefulness and negatively with NREM and REM sleep [32], whether measured during periods of sleep deprivation or following the use of stimulants such as modafinil [17,101]. Thus, excitatory orexin input may help regulate normal cortical arousal and wakefulness. 

Nevertheless, orexin/hypocretin-deficient mammals do not exhibit excess sleep. Instead, their on-off circuitry appears unstable, which leads to poor sleep-wake maintenance and dysfunctional switching [98]. Mochizuki *et al.* confirmed that the state instability during wakefulness experienced by orexin-deficient mice is not a result of abnormal sleep homeostasis, poor circadian control, or defective monoaminergic systems but rather is the consequence of behavioral state instability due to low between-state transitional thresholds [68]. In short, the asymmetric relationship between orexin/hypocretin neurons and arousal and sleep-promoting cell populations may provide stability to the sleep-wake system by anchoring the flip-flop switch [99].

## HOMEOSTATIC REGULATION OF SLEEP

Sleep is understood to be restorative, but precisely what is being restored is uncertain. As a homeostatic process, sleep allows the body to return to equilibrium when it is disturbed. For instance, sleep deprivation tends to be followed by extra compensatory sleep to make up for the loss, albeit not on a minute-for-minute basis. Borbely and colleagues proposed a two-process model of sleep regulation to explain the homeostatic and circadian drives for sleep [1,12]. The homeostatic component, named Process S (sleep), is believed to derive from a substrate or protein that registers a homeostatic “need to sleep” during periods of extended wakefulness that is subsequently relieved during sleep. As NREM sleep appears to take precedence over REM sleep following acute sleep loss, it is probable that the homeostatic mechanisms for the two sleep states differ [99].

The underlying mechanisms remain unclear. However, McCarley and colleagues have shown that adenosine acting in the basal forebrain is a key mediator of homeostatic control (for a more detailed review, see McCarley 2007 [56]). Increased adenosine release accompanies the accumulation of the need to sleep, suggesting that the nucleoside, adenosine, may be involved in the homeostatic control of sleep expression [9]. During periods of wakefulness, glycogen, the body’s principal store of energy, is exhausted [47]. As glycogen is broken down into adenosine, extracellular levels of adenosine begin to accumulate in the basal forebrain [87], leading to the replenishment of glycogen levels with recovered sleep [47,105]. Experimental models showed that the injection of adenosine or an adenosine A1 receptor agonist into the rat basal forebrain or the cat VLPO, respectively, promoted sleep by inhibiting multiple wake-promoting regions of the brain or exciting sleep-promoting cell groups [101,112]. Adenosine also may excite VLPO neurons by disinhibiting GABAergic inputs [16]. Therefore, by inhibiting the basal forebrain arousal system and triggering the VLPO nucleus, adenosine may act as homeostatic regulator of the sleep need. Recent evidence has shown that the sleep-promoting effects of adenosine are further enhanced through its action at the A1 receptor, which triggers an intracellular cascade leading to increased adenosine A1 receptor production [56]. Other mediators of homeostatic drive may be identified in the future. 

## CIRCADIAN SLEEP REGULATION

A second component of the sleep-wake regulatory mechanism, which Borbely called Process C, involves circadian influences [1,12]. Dijk and Czeisler confirmed the role of the circadian pacemaker in the timing of the sleep-wake cycle and regulation of the internal structure of sleep in a study in which 8 men lived in an environment free of time cues [27]. They found that sleep propensity and sleep structure derive from the interactions of circadian and sleep-wake-dependent oscillatory processes. The locus of this endogenous circadian pacemaker is the suprachiasmatic nucleus (SCN) of the hypothalamus.

The SCN, which directs the circadian program, has been called the brain’s “master clock” [89]. Circadian timing, in which neurons fire in a 24-hour cycle, is organized in a hierarchy of tissue-specific structures located throughout the body. These tissue-specific rhythms are coordinated by the SCN based on light input from the outside world during daytime and by melatonin secretion during the dark cycle [15]. Damage to the SCN eliminates the circadian rhythms of many behaviors, including sleep [69]. In particular, lesions of the retinohypothalamic tract (RHT) of the SCN, which processes light input, cause animals to exhibit free-running behaviors, demonstrating that the SCN is necessary for synchronization of circadian rhythms to the solar day [44].

Early studies of circadian rhythms in rats with lesions of the SCN suggested that the homeostatic process was independent of the circadian clock, as disrupted circadian sleep-wake cycles following SCN destruction were not accompanied by changes in overall sleep duration or recovery after sleep [64,65]. In 1993, however, Edgar and colleagues found that lesions of the primate SCN caused both a loss of circadian timing and increased total sleep time, suggesting an interaction of circadian and homeostatic processes. According to Edgar’s “opponent process” model, the SCN master circadian clock produces an alerting signal to enhance wakefulness and to actively counteract the accumulation of homeostatic drive for sleep [30].

A number of recent experimental studies have clarified the relationship between the SCN and the sleep-wake cycle. Most outputs from the SCN are directed at the subparaventricular zone (SPZ) and dorsomedial nucleus of the hypothalamus (DMH); in contrast, there is little innervation of the VLPO or orexin neurons [95,128]. Lu and colleagues showed that, in rats, lesions of the ventral SPZ disrupted measures of the circadian index of sleep by 90% but had little effect on body temperature; whereas, dorsal SPZ lesions lowered the circadian rhythms of temperature by 75% but did not affect sleep (<5%) [53]. This finding indicates that the SPZ is a complex region comprising neuronal subpopulations that differentially regulate circadian rhythms of different physiological responses. 

Moreover, the SPZ appears to link circadian input from the SCN to the DMH and preoptic targets, thereby amplifying the circadian responses [53]. The DMH is a particularly important conduit for delivering signals from the SCN to the sleep-regulatory system. Lesions of the DMH reduced circadian rhythms of wakefulness, feeding, locomotor activity, and serum corticosteroid levels by 78% to 89% [19]. The DMH also sends GABAergic projections to the sleep-promoting VLPO nucleus and glutamate-thyrotropin-releasing hormone afferents to the excitatory lateral hypothalamic area [19]. Therefore, by integrating clock information from the SCN and the SPZ, the DMH plays a major role in regulating circadian sleep behavior.

**Fig. (4):**
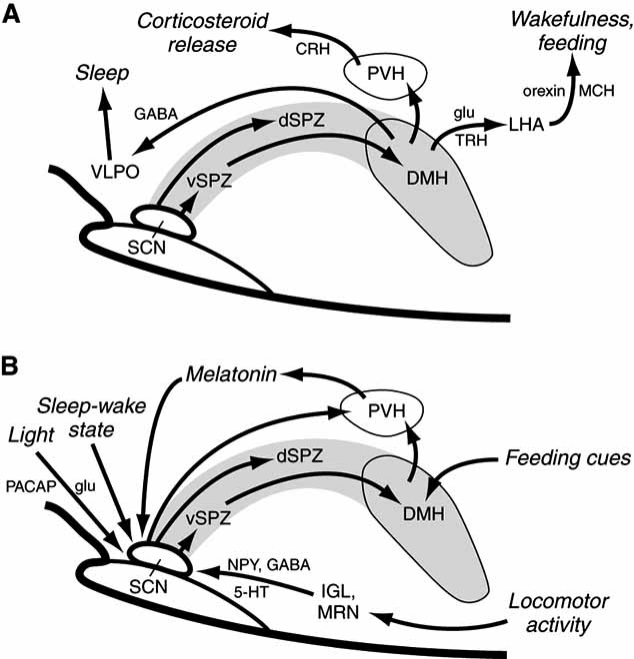
Circadian regulation of sleep-wake cycles. Adapted from Fuller 2006, pg 488 [33].

In sum, an intricate multistage pathway connecting the SCN, the ascending arousal system, and the VLPO *via* the SPZ and DMH regulates circadian rhythms of sleep and other behaviors (Fig. **4**). One rationale for this complexity is that it gives mammals the flexibility to adapt their behavioral and physiological cycles to environmental cues, and therefore establish patterns of rest-activity and sleep-wakefulness that are best able to meet the organism’s needs [97,99]. For instance, hypothalamic orexin neurons monitor indices of energy balance and mediate adaptive arousal due to food scarcity [127]. Whereas orexin expression in normal mice decreases as levels of blood glucose and leptin rise following food intake, mice with ablated orexin neurons do not respond to fasting with increased arousal and food-seeking activity. Precisely how external forces such as food availability and predation are able to reset homeostatic and circadian systems is currently unknown. 

## THE NEUROPATHOLOGY OF SLEEP-WAKE DISORDERS

At present, the pathophysiology of many sleep-wake disorders is poorly understood [41]. Generally, a combination of biological, psychological, and social factors is implicated in the etiology of these conditions. The remainder of this review will describe the substrates and mechanisms that have been identified in the most common sleep-wake disorders and the clinical implications for the selection of suitable treatment strategies.

### Insomnia

Insomnia, the most frequently reported sleep disorder, is characterized as a state of hyperarousal in which stress is believed to activate the hypothalamic-pituitary-adrenal axis [41,85]. Vgontzas *et al.* demonstrated that, compared with healthy subjects, those with chronic insomnia had increased secretion of corticotropin and cortisol throughout the sleep-wake cycle [121]. Additionally, Nofzinger and colleagues, using positron emission tomography (PET) studies to assess regional cerebral glucose metabolism, demonstrated that insomnia also is associated with greater whole-brain metabolism during both sleep and wake periods and, notably, a failure of wake-promoting structures to deactivate during the transition from waking to sleep states [78]. Structures regulating the sleep-wake cycle, such as the brainstem, hypothalamus, and basal forebrain, are abnormally overactive during sleep. The ventral emotional neural system also is hyperactive during wakefulness in patients with primary insomnia and insomnia associated with depression, and this abnormal activity persists into NREM sleep [77]. These PET findings of whole brain hypermetabolism during sleep and wake states, and reduced waking metabolism in the prefrontal cortex of patients with insomnia, suggest that they have chronic insufficient sleep, which may explain daytime symptoms of fatigue [78]. The results also may explain why cognitive factors (eg, worry) and environmental cues (eg, light exposure and unstable sleep schedules) perpetuate insomnia. A recent review by Roth and colleagues explores the pathophysiology of insomnia and treatment implications in more detail [91]. For more information about treatment for insomnia, see Morin 2005 [71].

### Narcolepsy

Narcolepsy is characterized by excessive daytime sleeping and abnormal REM sleep [60,100]. Little is known about the cause of the disorder, and the little that is understood pertains to narcolepsy with cataplexy [23]. As noted above, individuals with this condition have marked neuronal loss (85% to 95%) in the hypothalamic regions responsible for producing orexin/hypocretin, including the dorsal and lateral hypothalamus, as well as in the locus coeruleus, thalamus, and the cerebral cortex [86,113]. The presence of gliosis in the orexin cell region suggests that narcolepsy is the consequence of a neurodegenerative process [113]. Although the precise mechanisms are unknown, it is probable that both genetic and environmental triggers are involved in the onset of the condition [60].

### Disorders Associated With Fragmented Sleep

Obstructive sleep apnea (OSA), the most prevalent type of sleep disordered breathing, is characterized by repeated episodes of partial (hypopneas) or complete collapse (apneas) of the pharyngeal airway [41]. The apneic or hypopneic episodes lead to arousal and may induce hypoxemia and hypercapnia, as well as abnormally high levels of sympathetic nervous system activity [74]; these neuropathological changes are mediated by chemoreceptors in the carotid body and brainstem. High levels of sympathetic drive are evident even during periods of wakefulness, when subjects are breathing normally and there is no evidence of chemoreceptor activation [74]. OSA is associated with hypertension, cardiovascular disease, and depression [80,84]. OSA patients have a higher incidence of stroke and transient ischemic attacks as well [76,80]. 

Another common disorder associated with fragmented sleep is restless legs syndrome (RLS). People with RLS report a strong urge to move the legs, although the arms, trunk, or head and neck may be affected as well [4]. Symptoms of paresthesias worsen at night and at rest, and are relieved by movement, making it difficult to fall asleep or maintain sleep [41]. Altered dopamine and iron metabolism has been proposed as a mechanism of RLS, and a strong familial component has been identified as well [125]. Subjects with RLS have low iron levels in the substantia nigra and putamen (regions of the brain that are responsible for controlling voluntary movement), and this disturbs the normal transmission of dopamine signals [3,116]. More specifically, Connor and colleagues suggested a defect in the regulation of transferrin receptors on neuromelanin-containing cells as a possible trigger [20]. The fact that RLS is common in individuals with iron deficiency provides clinical support for this hypothesis [41]. In addition, dopaminergic agonists have been shown to be effective in the treatment of RLS, whereas dopamine antagonists aggravate sensorimotor symptoms and sleep disturbances [123,124,126]. Taken together, these findings demonstrate that impaired iron metabolism is involved in the pathogenesis of RLS; whether other causative factors are involved is still to be determined [3].

### Circadian Rhythm Sleep Disorders

Circadian rhythm sleep disorders stem from a chronic disturbance in the relationship between the circadian pacemaker and environmental cues (eg, the light-dark cycle) [41]. In their 2005 ICSD update, the American Academy of Sleep Disorders identified 9 circadian disorders, including delayed sleep phase type, advanced sleep phase type, shift work type, nonentrained sleep-wake type, irregular sleep-wake type, and jet lag type [5]. The most prevalent of these conditions is shift work type circadian rhythm sleep disorder, also known as shift work disorder (SWD).

Shift work disorder is characterized by complaints of insomnia or excessive sleepiness (ES) during work hours scheduled during the customary sleep periods [5]; it is estimated to affect about 32% of night-shift workers and 26% of rotating-shift workers [29]. It is believed to be caused by misalignment of circadian regulation and sleep-wake behavior (ie, displaced work hours) [2]. Several different kinds of shift-work schedules may be implicated in the etiology of SWD – night shifts, early morning shifts, and rotating shifts, with the first two predominating. In people with SWD, total sleep time is reduced by 1 to 4 hours, and sleep quality is described as unsatisfactory. Patients may also complain of difficulties with sleep initiation and awakening; ES and diminished alertness, which may impair mental ability and work performance, also are hallmarks of SWD. The ES is due in part to total sleep loss as well as to a decreased circadian alerting signal that corresponds to the altered work hours [5]. 

The pathology of SWD raises important safety concerns, particularly with respect to the elevated risk of motor vehicle accidents and workplace injury [79]. People with SWD also have higher rates of cardiovascular and gastrointestinal disease and depression, and are more likely to miss family and social activities than are shift workers without the disorder [29]. See Schwartz and Roth 2006 for a more complete review of the burden of illness and management approaches to SWD [104]. 

### Disordered Sleep Associated with Primary Neurological Disorders: Alzheimer’s and Parkinson’s Diseases

Neurodegeneration in brain regions that are involved in sleep regulation can produce sleep-pattern abnormalities. Although the pathogenesis of sleep disturbances associated with Alzheimer’s disease (AD) is unknown [21,25], a relationship between disordered sleep and a number of behavioral disturbances associated with dementia, such as aggressiveness and depression, has been identified [70,114]. Individuals with AD also have a higher prevalence of OSA [11], possibly because both conditions are associated with the APOE4 allele. 

In contrast, the etiology of Parkinson’s disease (PD)-related sleep disorders is better established. (Askenasy 2003 provides a useful introduction [7].) PD is a dopaminergic disease, and patients with PD have fewer dopaminergic neurons in cell groups of the ascending arousal system, relative to controls [129]. Other loci of arousal, such as noradrenergic and cholinergic nuclei, are depleted in PD as well [41]. There also is evidence that Lewy body degeneration in the lower brainstem, substantia nigra, and other mid- and forebrain gray matter regions begins relatively early in the disease process [13]. This finding is consistent with the observation that REM sleep disturbances are common in PD and may precede the onset of overt symptoms [13,41]. Despite these insights, much about the neurobiology of disordered sleep secondary to AD, PD, and other primary neurodegenerative conditions remains to be described. 

### Treatment Considerations

Sleep disturbances are typically treated with a combination of behavioral and pharmacological therapies. The former include various psychological techniques such as cognitive behavioral and sleep-restriction therapy, and strategies to improve sleep hygiene. Pharmacotherapeutic approaches include hypnotic agents to improve sleep onset and maintenance, and wake-promoting agents such as sympathomimetic alerting agents (such as amphetamines and methylphenidate) and modafinil. Other therapies that have been beneficial in primary sleep-wake disorders include continuous positive airway pressure (CPAP) for OSA, antidepressants and sodium oxybate for narcolepsy with cataplexy, and benzodiazepine receptor agonists and melatonin selective receptor agonists for insomnia. Dopamine agonists, benzodiazepines, opioids, and anticonvulsants (including gabapentin) are prescribed for RLS; all may be effective in treating various RLS-related symptoms. The dopamine agonists ropinirole and pramipexole have become first-line agents approved for the treatment of mild-to-moderate RLS.

Sleep problems secondary to neurological disease can be more complex. While AD patients with disturbed sleep should have the underlying symptoms addressed in the same manner as patients without dementia, treatment of PD-related sleep disturbances is complicated by the fact that dopaminergic medications used to treat the primary condition may cause nocturnal wakefulness, decreased short-wave sleep, and decreased sleep continuity at high doses [41]. Paradoxically, dopamine agonists may also cause ES [83], requiring the addition of a wake-promoting medication for symptomatic relief. 

### Caffeine

Caffeine is often used as the initial treatment for ES caused by sleep deprivation. It achieves its wake-promoting effects by antagonizing adenosinergic neurons located in the hypothalamus and projecting into cells in the cortex, basal forebrain, and reticular activating system [67]. As described previously, endogenous adenosine levels rise as the need for sleep builds [9]. By inhibiting the basal forebrain arousal system and activating the VLPO, adenosine appears to be a fundamental component in the regulation of the homeostatic sleep system. Through its inhibition of A1 adenosine receptors, caffeine prevents sleep onset and maintenance. 

It is well established that caffeine affects alertness and cognitive performance, and may reduce the risk of accident and injury in sleep-deprived individuals. Caffeine supplements in doses of 200 mg improved psychomotor performance tasks and vigilance in young, healthy, non-caffeine dependent individuals [73]. However, tolerance can rapidly develop, and there is no long-term evidence validating its use. Caffeine is not indicated for use in narcolepsy, OSA, SWD, or idiopathic hypersomnia. 

### Sympathomimetic Alerting Drugs

The mechanism of action of sympathomimetic alerting drugs (eg, dextro- and methamphetamine, methylphenidate) is direct or indirect stimulation of dopaminergic and noradrenergic nuclei, which in turn heightens the efficacy of the ventral periaqueductal grey area and locus coeruleus, both components of the secondary branch of the ascending arousal system. Amphetamines also activate non-wake-promoting CNS regions, such as the striatum and nucleus accumbens, causing the adverse effects (eg, nervousness, irritability, anorexia, gastrointestinal problems, and rebound hypersomnia), seen with this class of wake-promoting drugs. In addition, amphetamines have a high abuse potential and can lead to dependence. Some patients may also develop tolerance to the alerting effects of these drugs, although the frequency varies across studies [81,82]. For these individuals, changing medications or introducing a drug “holiday” can improve the response [10].

Sympathomimetic drugs have long been used to treat narcolepsy, although many patients find that amphetamines do not provide a sufficient degree of daytime alertness [66]. For these patients, combining 2 or more alerting drugs (eg, sodium oxybate [see below]) can be beneficial. Amphetamines are available in a wide range of formulations and half-lives to offer flexibility in dosing. The daily dose range for methylphenidate is up to 60 mg [90]; dextroamphetamine is up to 60 mg; and methamphetamine is 50 mg [38]. Amphetamines carry a relative contraindication for those with pre-existing cardiovascular problems, including hypertension. For all patients, regular blood pressure monitoring is recommended. 

### Antidepressants and Sodium Oxybate as Anticataplectic Drugs

Cataplexy is usually treated with tricyclic antidepressants (TCAs), selective serotonin reuptake inhibitors (SSRIs) or norepinephrine reuptake inhibitors (NRIs) [41]. Newer antidepressants that are not TCAs or SSRIs, such as atomoxetine and venlafaxine, also may be effective [10]. In addition, patients may benefit from sodium oxybate. Although the mechanism of sodium oxybate has been studied extensively, it remains unknown [115]. The treatment is especially effective when combined with other alerting drugs and has been shown to reduce nocturnal sleep disruptions and help consolidate sleep [10].

### Modafinil and Armodafinil

Modafinil is a unique wake-promoting compound that is pharmacologically and chemically distinct from other CNS stimulants. A substantial body of research has sought to elucidate the precise mechanism of action of modafinil; to date, the question remains unresolved. (See Schwartz 2005 for a detailed review of the pharmacology of the drug [102].) Preclinical studies show that modafinil has low affinity for receptors for noradrenaline, serotonin, GABA, adenosine, or histamine [26,62,108]. However, Scammell and colleagues reported that modafinil increased activity in the TMN and orexin nuclei, 2 regions involved in the promotion of wakefulness [101]. The drug also potentiates noradrenergic nuclei in the VLPO, thereby inhibiting sleep [35]. Consequently, it is believed that by down-regulating sleep-promoting neurons in the VLPO, modafinil enables components of the ascending arousal neuronal pathway (eg, the TMN) to remain active [102]. Further research will be necessary to confirm this hypothesis.

Modafinil is approved for the treatment of ES associated with narcolepsy, OSA, and SWD. Multiple randomized clinical trials in these 3 conditions demonstrated that modafinil produced significant improvement, relative to placebo, in subjective and objective measures of ES, including the Epworth Sleepiness Scale, Multiple Sleep Latency Test, and Maintenance of Wakefulness Test [22,28, 117,118]. Overall clinical condition and sustained attention and reaction time, as measured by the Clinical Global Impression of Change and Psychomotor Vigilance Test, respectively, also improved significantly. In addition, patients with SWD given modafinil reported a significantly lower rate of driving accidents or near accidents while commuting [22]. The drug was well tolerated in these clinical trials, with mild-to-moderate, transient headache being the primary reported adverse event [22,28,117,118]. There also have been cases of transient insomnia. Overall, modafinil has a superior side effect profile compared with other wake-promoting drugs, and has a low potential for abuse. For these reasons, the agent has become a treatment of choice for narcolepsy, and is the only currently available medication that is approved for OSA patients treated with CPAP with ongoing daytime ES, as well as patients with SWD [102]. The standard dose is 200 to 400 mg daily [88], although some patients with severe sleepiness may require higher doses, and doses up to 600 mg in divided doses have shown a subjective and objective benefit over lower doses in narcolepsy patients (400 mg at 7 am, 200 mg at noon [103]. 

Armodafinil is the R-isomer of modafinil and is approved for treatment of ES associated with OSA, SWD, and narcolepsy. In clinical trials, armodafinil has been shown to improve wakefulness throughout the day in patients with OSA or narcolepsy [41] and during the night shift and the commute home in patients with SWD [30]. The most common adverse events reported in clinical studies of armodafinil were headache, nausea, and insomnia.

### Benzodiazepine Receptor and Melatonin Selective Receptor Agonists 

Treatment of insomnia typically involves a combination of behavioral and pharmacological approaches. Among the psychological techniques that have demonstrated effectiveness are stimulus-control and sleep-restriction therapies, relaxation training, cognitive behavioral therapy, and sleep hygiene education [69]. The most frequently prescribed pharmacotherapies for insomnia are GABA type A (GABA-A) modulators. These drugs target the benzodiazepine receptors in the CNS. Benzodiazepine (eg, temazepam, triazolam) and non-benzodiazepine (zolpidem, eszopiclone) hypnotics may produce daytime sedation side effects in some patients, but these problems do not limit their utility. Benzodiazepine receptor agonists have an abuse and dependence potential (Schedule IV), especially in patients with a history of substance abuse. Careful surveillance of at-risk individuals is recommended. Benzodiazepine hypnotics also may cause next-day cognitive and motor impairments [111,120] and may engender withdrawal symptoms (eg, rebound insomnia, anxiety, irritability, gastrointestinal distress), particularly following long-term or high-dose use [39]. 

The recently approved MT1/MT2 melatonin receptor agonist ramelteon is a proposed alternative to the benzodiazepine receptor agonists for the treatment of insomnia characterized by difficulty with sleep onset [93]. MT1 and MT2 receptors are located in the SCN and contribute to maintenance of the circadian sleep-wake cycle. As the drug has no affinity for benzodiazepine, dopamine, and opiate receptors, or for ion channels and receptor transporters, ramelteon has limited potential for abuse and cognitive and functional impairment. A dose-ranging, double-blind, cross-over study comparing ramelteon, triazolam, and placebo found that, compared with placebo, ramelteon caused no significant effect on these problematic adverse events at up to 20 times the recommended dose (8 mg daily at bedtime) [43]. In contrast, triazolam negatively affected measures of motor and cognitive performance. Ramelteon significantly reduced latency of persistent sleep and increased total sleep time in adults with chronic insomnia in 2 double-blind randomized, controlled trails [31,92]. There also were no apparent next-day cognitive or motor effects or evidence of rebound insomnia or withdrawal effects following treatment discontinuation in these trials. Ramelteon should be considered for patients with sleep-onset insomnia, particularly those who are treatment naïve, who have a history of substance abuse, who are older adults susceptible to the effects of benzodiazepine and non-benzodiazepine hypnotics, and who require minimal interference with the arousal response. 

## CONCLUSION

Recent insight into the physiological patterns of sleep and wakefulness has shown that different brain-processing networks and neurochemical systems are involved in both states. The particular neuronal pathways, transmitters, and receptors that make up the ascending arousal system centered in the hypothalamus interact with sleep-active neurons in the VLPO in a flip-flop switch to produce distinct sleep-wake states with abrupt transitions. Progress in understanding the neural circuitry underlying the regulation of sleep-wake states has led to the identification of new mechanisms and substrates, and studies are now underway to investigate these potential targets. In the future, the products of these investigations may offer novel approaches for the treatment of these common and intractable conditions.
